# Outcomes of ventilated surgical and medical ICU patients: do patients die from ARDS or with ARDS?

**DOI:** 10.1186/cc13219

**Published:** 2014-03-17

**Authors:** L Fuchs, D Talmor

**Affiliations:** 1 Soroka University Medical Center, Beer Sheba, Israel; 2 Beth Israel Deaconess Medical Center, Boston, USA

## Introduction

Patients with acute respiratory distress syndrome (ARDS) have a high mortality rate. Whether this excess mortality is contributed by ARDS or is a consequence of prolonged mechanical ventilation is unclear.

## Methods

A longitudinal retrospective study focusing on noncardiac ICU patients who required mechanical ventilation. Patients were classified as having ARDS on admission, late-onset ARDS or no ARDS. The analysis included patients ventilated for longer than 48 hours. Primary outcomes were mortality at 28 days, 1 year and 2 years from ICU admission.

## Results

A total of 1,396 patients were enrolled between 2001 and 2008: 485 (34.7%) had ARDS on admission (early-onset ARDS), 219 (15.6%) developed ARDS during their ICU stay (late-onset ARDS) and 692 (49.5%) did not meet ARDS criteria prior to ICU discharge or death. Twenty-eight-day mortality rates were 23.7%, 25.6% and 25.7% for early, late and non-ARDS respectively. After adjusting for age, weight on admission, unit of admission (MICU vs. SICU), severity of disease score, comorbidity score, and primary diagnosis on admission, and mortality risk at 28 days, 1 year and 2 years were not significantly different between the three study groups. Neither severity of ARDS or timing of ARDS (early versus late) was associated with mortality. Sensitivity analysis, analyzing all ventilated patients, including those who were ventilated less than 48 hours, showed the same results.

## Conclusion

Neither the presence of ARDS or the severity or timing contribute independently to the short and long mortality risk after adjustment for age, severity of disease, comorbidity score and diagnosis on presentation in patients hospitalized in a noncardiac ICU with acute respiratory failure.

